# Sitagliptin attenuates high glucose-induced alterations in migration, proliferation, calcification and apoptosis of vascular smooth muscle cells through ERK1/2 signal pathway

**DOI:** 10.18632/oncotarget.20417

**Published:** 2017-08-24

**Authors:** Lili Shi, Ye Ji, Dandan Liu, Ying Liu, Ying Xu, Yang Cao, Xiaoyan Jiang, Changqing Xu

**Affiliations:** ^1^ Department of Endocrinology, The First Affiliated Hospital of Harbin Medical University, Harbin, Heilongjiang 150081, P.R. China; ^2^ Department of Orthopedics, The Second Affiliated Hospital of Harbin Medical University, Harbin, Heilongjiang 150081, P.R. China; ^3^ Department of Pathophysiology, Harbin Medical University, Harbin, Heilongjiang 150081, P.R. China

**Keywords:** vascular smooth muscle cells, atherosclerosis, diabetes, high glucose, dipeptidyl peptidase-4 inhibitor

## Abstract

**Background/Aims:**

This study investigated the effects of sitagliptin on migration, proliferation, calcification and apoptosis of vascular smooth muscle cells (VSMCs) under high glucose (HG) conditions.

**Methods:**

VSMCs were isolated from the thoracic aorta of Sprague Dawley rats. The cultured VSMCs were subjected to control medium, mannitol medium, HG medium (25 mM), pretreatment with 200 nM sitagliptin in control or HG medium, or the ERK1/2 inhibitor PD98059 in HG medium. Cell proliferation, migration, apoptosis and calcification were determined.

**Results:**

HG conditions promoted the proliferation, migration, calcification and impairment of apoptosis in VSMCs compared with controls (P<0.05). Pretreatment with sitagliptin effectively attenuated proliferation, migration, calcification of cells and increased apoptosis of HG-cultured VSMCs as compared with the HG group (P<0.05). Culture with HG resulted in the up-regulation of p-ERK1/2 in VSMCs, whereas sitagliptin pretreatment could inhibit HG-induced p-ERK1/2 expression. In addition, the ERK1/2 inhibitor PD98059, inhibited proliferation, migration, calcification and promoted the apoptosis of HG-cultured VSMCs compared with the HG group (P<0.05).

**Conclusion:**

The effects of sitagliptin on VSMC under high glucose condition were achieved through ERK1/2 signaling pathways.

## INTRODUCTION

Atherosclerosis (AS) is one of the most common cardiovascular complications of diabetes, and remains the leading cause of death in diabetic patients [1]. The pathogenesis of atherosclerosis in diabetes is a complex process, involving endothelial cell dysfunction, lipid deposition, and recruitment of monocytes, as well as production of macrophage-derived foam cells. In addition, pathological changes of vascular smooth muscle cells (VSMCs) are also involved in the development atherosclerosis [2]. Accumulation of VSMCs in the arterial intima is the key process of atherosclerosis formation. Chronic hyperglycemia leads to increased production of reactive oxygen species (ROS), thus stimulating the proliferation of VSMCs and migration to arterial intima [3, 4]. It has been reported that hyperglycemia may inhibit apoptosis in VSMCs through the up-regulation of Bcl-2, Bcl-xL and *Bfl*-*1*/*A1*[5, 6]. Excessive proliferation and impaired apoptosis of VSMCs contribute to massive deposition of VSMCs in the vascular intima and medial atherosclerotic plaque [5], thereby leading to atherosclerotic vascular remodeling. Moreover, vascular calcification occurs in the presence of hyperglycemia [6]. Calcified VSMCs may undergo osteochondrocytic or osteocytic changes, resulting in atherosclerotic calcification, an adverse event associated with an increased risk of cardiovascular morbidity and mortality [7–9]. As excessive migration, proliferation, calcification and a reduction in apoptosis of VSMCs under hyperglycemic conditions are involved in the formation of atherosclerotic plaque formation in diabetes mellitus [3–6, 10], it is possible to treat diabetic atherosclerosis by inhibiting hyperglycemia-triggered events in VSMCs.

Dipeptidyl peptidase-4 (DPP-4) inhibitors are a new class of anti-diabetic drugs. They inhibit the activity of the DPP-4 enzyme, thereby preventing inactivation of glucagon-like peptide 1 (GLP-1) and increasing active GLP-1 concentrations in the blood, as well as its duration of action [11, 12]. Besides hypoglycemic effects, DPP4 inhibitors have been found to have both cerebro- and cardiovascular protective capabilities. Administration of sitagliptin at 50 or 100 mg twice daily for five days can effectively decrease mild to moderate non-diabetic hypertension [13]. DPP-4 inhibitors can decrease the incidence of atherosclerosis [14] and reduce infarct size [15], improve heart failure [15], and improve cognitive function [16]. Furthermore, it has been reported that sitagliptin treatment for six months can significantly improve cognitive function among diabetic patients with Alzheimer's disease [16]. In addition to these findings, treatment with sitagliptin exerts a protective effect against atherosclerosis, most likely through improvement of endothelial function, attenuation of inflammation and oxidative stress, reduced recruitment of monocytes and chemotaxis, as well as a decrease in plaque macrophage content [10–12, 14, 17, 18]. However, the exact mechanism by which sitagliptin reduces atherosclerotic lesions is complex and remains unclear. It is well known that the MAPK pathway is important for the regulation of cell migration, proliferation and apoptosis [19] and the ERK1/2 cascade functions to promote cellular proliferation, differentiation and survival. It has been reported that inhibition of the p38-MAPK signaling pathway prevents the occurrence of atherosclerotic disease [20–22]. Therefore, further investigation is needed to examine whether sitagliptin exerts its anti-atherosclerotic effects by, or partially, through action on ERK1/2-MAPK pathways.

As VSMCs are involved in the pathogenesis of cerebro-cardiovascular diseases, we speculated that the protective effect of DPP-4 inhibitors against atherosclerosis, AMI, heart failure may be through interaction with VSMCs. At present, anagliptin, another DPP-4 inhibitor, has been proven to reduce atherosclerotic lesions and inhibit the proliferation of VSMCs in apo E-deficient mice, which is achieved through the inhibition of ERK phosphorylation [23]. Currently, there are few studies exploring the effects of sitagliptin on the biological characteristics of VSMCs under high glucose (HG) condition. Therefore, this study investigated the effects of sitagliptin on VSMC migration, proliferation, calcification and apoptosis under HG condition, and explored the molecular mechanisms of sitagliptin in diabetic atherosclerosis.

## RESULTS

### Sitagliptin inhibited the HG-induced proliferation in VSMCs

**Figure 1:**
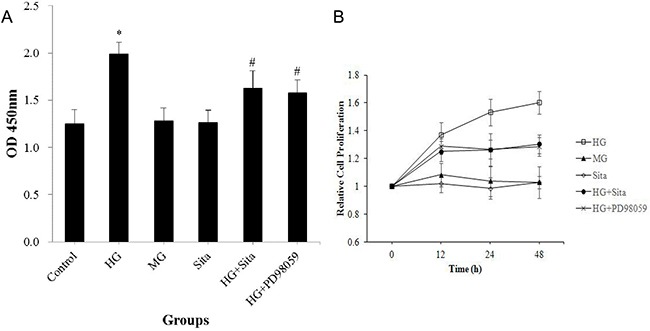
Sitagliptin inhibited the HG-induced proliferation in VSMCs **(A)** Cell proliferation was determined at 12, 24, 48 hours using a cell counting kit. **(B)** VSMC proliferation with different interventions and at 12, 24, 48 hours. All results are presented as mean ± SD from three independent experiments; *p<0.01 vs. control; #p<0.01 vs. HG. OD, optical density; HG, high glucose; MG, mannitol group; Sita, Sitagliptin.

After intervention for 12 h, 24 h and 48 h, HG, but not mannitol, remarkably promoted the proliferation of VSMCs as compared with the control (*P*<0.01, *vs.* control, Figure 1A). VSMCs proliferated with prolonged culture, detected by the cell counting kit-8 (Figure 1B). Sitagliptin treatment effectively inhibited the HG-induced proliferation in VSMCs (*P*<0.01, *vs.* HG group). However, sitagliptin alone did not influence the growth of VSMCs in normal condition (P>0.05, Figure 1A).

### Sitagliptin inhibited the HG-induced migration in VSMCs

**Figure 2:**
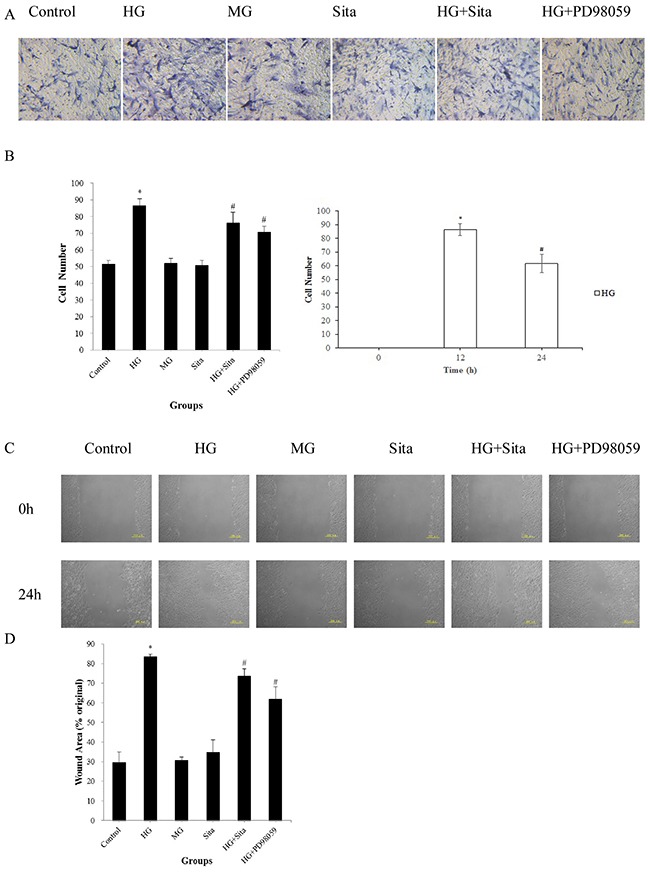
Sitagliptin inhibited HG-induced migration in cultured VSMCs **(A)** Transwell filters were stained at 24 h with hematoxylin to visualize migrated cells (200× magnification). **(B)** Left: Quantitation of migrated VSMCs in the Transwell migration assay; Right: Migrated VSMCs in the Transwell migration assay at different time points. *p<0.01 *vs.* control; #p<0.01 *vs.* HG. **(C)** Confluent VSMCs were wounded using a 200 μL sterile pipette tip and each scratch was observed at 0 and 24 h after wounding. The wound gaps in each culture were measured to indicate the migration capacity of VSMCs. **(D)** Quantification of the wound area in the scratch assays; *p<0.01 *vs.* control; #p<0.05 *vs.* HG. The results are presented as mean ± SD from three independent experiments. HG, high glucose; MG, mannitol group; Sita, Sitagliptin.

The migration capacity of VSMCs were measured by the Transwell assay and *in vitro* scratch assay (Figure 2). After culture with HG for 24 h, the number of migrated VSMCs on the underside of the Transwell filter was increased in comparison with the control (*P*<0.01, *vs.* control, Figure 2A). Moreover, VSMCs exhibited an enhanced migration capacity compared with controls (*P*<0.01, *vs.* control, Figure 2B left panel). In particular, HG promoted migration at 12-24 h compared to 0-12 h (P<0.01, Figure 2B right panel). Sitagliptin effectively inhibited the HG-induced migration in VSMCs, as evidenced by the decreased number of VSMCs on the Transwell filter, and reduced the wound area in the scratch assays (*P*<0.01 *vs.* HG group, Figure 2C-2D). There were no significant differences in migration capacity among the MG, SITA and control groups (*P*>0.05).

### Sitagliptin attenuated the inhibitory effects of HG on apoptosis of cultured VSMCs

**Figure 3:**
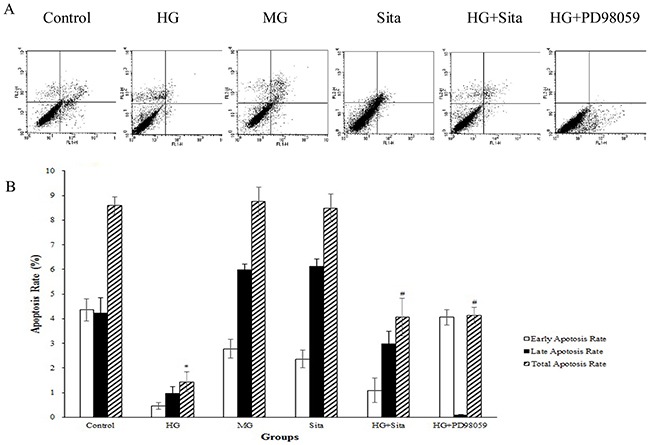
Sitagliptin (Sita) attenuated the inhibitory effects of HG on apoptosis of cultured VSMCs **(A)** Apoptosis was determined at 48 h by staining with Annexin V-FITC (X-axis) and propidium iodide (Y-axis). For each dot plot, the upper and lower right quadrants represent early apoptotic and late apoptotic cells, respectively. **(B)** Quantification of the apoptotic cells. Total apoptosis refers to the sum of early and late apoptosis values. Results are expressed as mean ± SD from three independent experiments; *p<0.01 *vs.* control; #p<0.05 *vs.* HG. HG, high glucose; MG, mannitol group. Sita, Sitagliptin.

Apoptosis was determined with Annexin V-FITC staining using flow cytometry (Figure 3). Compared with the control, culture with HG for 48 h reduced the apoptosis of cultured VSMCs (*P*<0.01 *vs.* control, Figure 3A-3B). Pretreatment with sitagliptin for 48 h potently attenuated the inhibitory effects of HG on apoptosis of cultured VSMCs (*P*<0.01 *vs.* HG group, Figure 3A-3B). However, mannitol and sitagliptin alone did not have any effect on apoptosis of cultured VSMC (*P*>0.05 *vs.* control).

### Sitagliptin inhibited HG-induced VSMC transition to osteoblast-like cells

**Figure 4:**
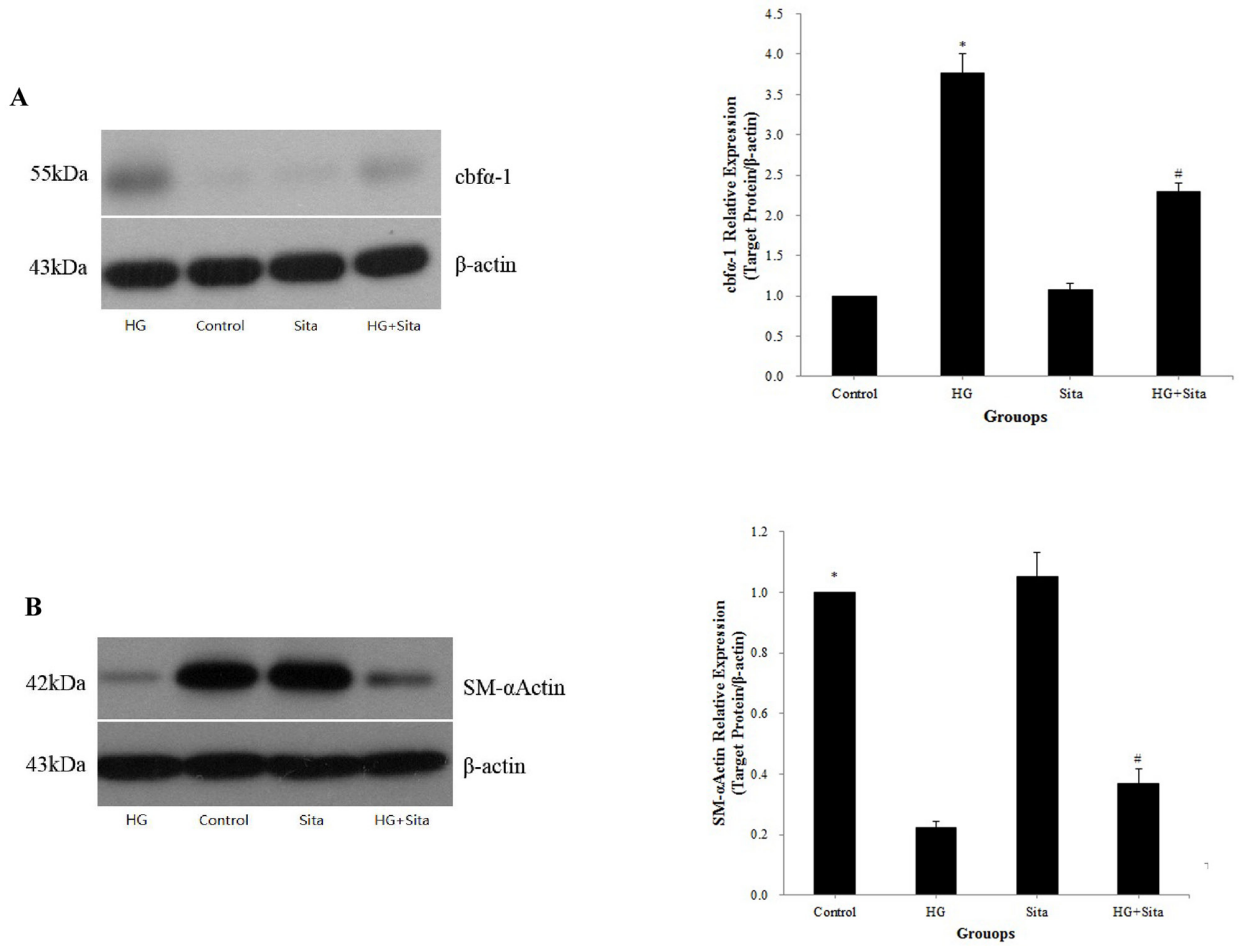
Sitagliptin reversed VSMC transition to osteoblast-like cells The expressions of the osteoblast-specific marker Cbfα-1 **(A)** and VSMCs marker SM-α-actin **(B)** were detected by Western blot analysis at two weeks after culture with HG medium. Data from three independent experiments are expressed as mean ± SD; *p<0.01 *vs.* control; #p<0.01 *vs.* HG. HG, high glucose; Sita, Sitagliptin.

The expression of the VSMCs marker smooth muscle α-actin (SM-α-actin) and osteoblast-specific marker core-binding factor α 1 (Cbfα-1) were detected by Western blot analysis (Figure 4). Compared with the control, HG triggered an increased expression of Cbfα-1 (P<0.01 *vs.* control, Figure 4A) and decreased the expression of SM-α-actin (P<0.01 *vs.* control, Figure 4B). Pretreatment with sitagliptin effectively reduced the expression of Cbfα-1 (Figure 4A) and increased the expression of SM-α-actin (Figure 4B) compared to the HG group (P <0.01 *vs.* HG group).

**Figure 5:**
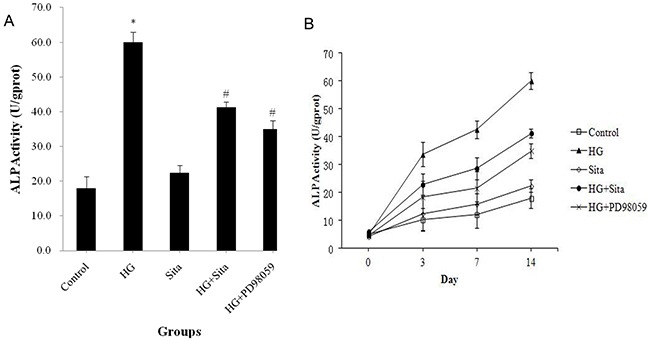
Sitagliptin reversed elevated ALP activity induced by HG in VSMCs ALP activity was measured using a ALP activity detection kit after culture with HG for 3, 7 and 14 days. **(A)** ALP activity after HG culture for 14 day. **(B)** ALP activity at different time points. *P<0.01 *vs.* control; #p<0.01 *vs.* HG. HG, high glucose; Sita, Sitagliptin.

ALP activity in cultured VSMCs was measured (Figure 5). HG culture resulted in an increase in ALP activity in a time-dependent manner (P<0.01 *vs.* control, Figure 5B). This effect could be reversed by sitagliptin pretreatment (P<0.01 *vs.* HG, Figure 5A).

**Figure 6:**
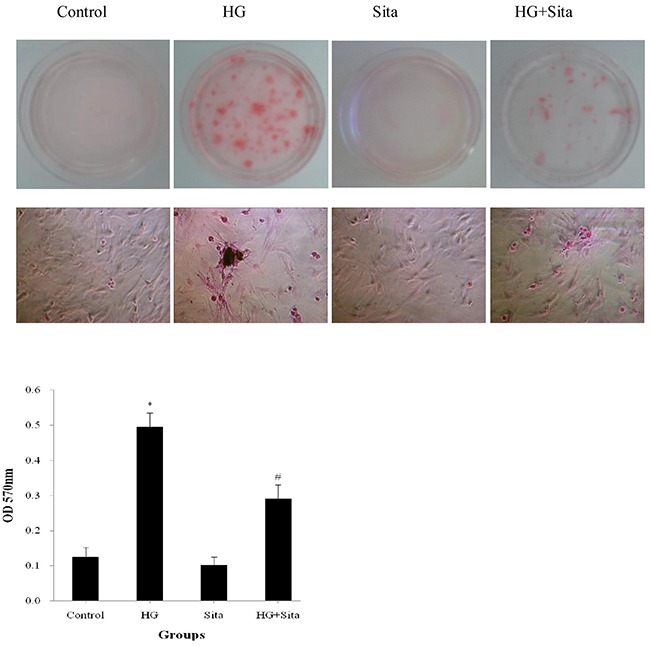
Sitagliptin (Sita) inhibited HG-induced calcification in VSMCs Calcification of cultured VSMCs was determined with alizarin red staining after HG culture for 14 days (400× magnification). *P<0.01 *vs.* control; #p<0.01 *vs.* HG. HG, high glucose; Sita, Sitagliptin.

In addition, the effect of sitagliptin on HG-induced cell calcification was tested with Alizarin Red staining (Figure 6). Compared with the control, VSMCs in HG group exhibited markedly increased alizarin red staining with the formation of calcified nodules (P<0.05 *vs.* control). Sitagliptin pretreatment resulted in a significant reduction in the amount of Alizarin Red staining, compared with HG group (P<0.05 *vs.* HG). There were no differences in the amount of Alizarin Red staining between SITA and control group. Taken together, HG promoted VSMC transition to osteoblast-like cells, thereby contributing to bone formation and calcification. These effects could be potently inhibited by sitagliptin pretreatment.

### Sitagliptin inhibited ERK1/2 MAPK signal pathways under HG conditions

**Figure 7:**
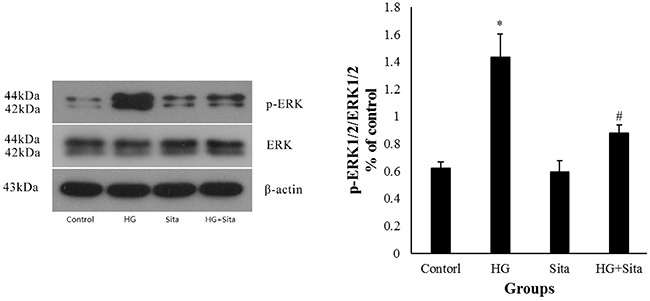
Sitagliptin inhibited ERK1/2 MAPK signal pathways under HG condition The expression of p-ERK1/2 in VSMCs was determined by Western blot analysis at 30 min after intervention. Data from three independent experiments are expressed as mean ± SD; *p<0.01 vs. control; #p< 0.01 vs. HG. HG, high glucose; Sita, Sitagliptin.

As shown in Figure 1-5, the ERK1/2 inhibitor PD98059 led to a decrease in proliferation, migration and calcification, and an increase in cell apoptosis in VSMCs under HG condition, compared with the control group (P<0.05 *vs.* control). HG culture significantly increased the expression level of p-ERK1/2 (*P*<0.01 vs. control), and sitagliptin pretreatment could inhibit HG-induced p-ERK1/2 expression (Figure 7). These data indicated that sitagliptin may exert an effect on HG-induced proliferation, migration, apoptosis and calcification, most likely through ERK1/2 MAPK signal pathways.

## DISCUSSION

This study for the first time provides evidence that sitagliptin may inhibit HG-induced alterations including proliferation, migration, apoptosis and calcification in cultured VSMCs, probably through the inhibition of ERK1/2 signaling pathways, revealing the favorable effects of sitagliptin for preventing the occurrence of vascular calcification and atherosclerosis in diabetes.

The changes in biological characteristics of VSMCs, including migration, proliferation, apoptosis and calcification, play an important role in the pathogenesis of diabetic atherosclerosis [24–26]. Previous studies have shown that VSMCs exhibited increased migration and proliferation [27, 28], along with a reduction in apoptosis under HG culture *in vitro* [29]. This study showed that high glucose promoted VSMC migration and proliferation, and inhibited apoptosis, which was consistent with previous findings. Vascular smooth muscle cell (VSMC) populations within the normal vascular media are responsible for maintaining vascular tone. Consequently, VSMCs appear in a contracted state, and are characterized by high expression of genes which encode α-SMA, SM22α and smooth muscle myosin heavy chain (SMM-HC) [30]. VSMCs in a contracted state are differentiated, mostly distributed in the middle layer of blood vessels or in the fibrous cap (FC) of atherosclerotic plaques [31]. Under certain factors, the VSMCs in the middle layer of blood vessels dedifferentiate from a “contractile phenotype into a “synthetic phenotype”. These cells migrate into the intima where they proliferate to repair damage. Furthermore, these cells can be differentiated into mesenchymal lineage cells such as osteoblasts, chondrocytes and adipocytes. The differentiation of VSMCs into osteoblasts leads to the occurrence of vascular calcification. Vascular calcification represents a crucial risk factor for adverse cardiovascular events, such as acute myocardial infarction and congestive heart failure. Previous studies have shown that VSMCs are involved in the formation of vascular calcification by undergoing a transition to an osteoblast-like phenotype and secreting matrix proteins [32, 33]. Hyperglycemia leads to elevated levels of circulating advanced glycation end products, participating in vascular calcification and atherosclerosis [34, 35]. Chen et *al*. found that culture with HG for 48 h induced expression of Cbfa-1 and bone morphogenetic protein-2 (BMP-2), a known osteoinductive factor, and significantly enhanced calcification in VSMC in a time-dependent manner [10]. Our study demonstrated that HG significantly increased the expression of Cbfα-1 and the activity of ALP, and prevented a decrease in the expression of the VSMC marker SMα-actin, suggesting that hyperglycaemia may directly induce vascular calcification through causing VSMC to transition to osteoblast-like cells, which was consistent with previous findings. MAPK signal proteins include ERK 1/2, p38, JNK/SAPK, ERK 3/4 and ERK 5[36]. MAPK signaling pathway is implicated in diabetes mellitus and atherosclerosis. Studies have shown that MAPK signaling pathway is associated with the migration and proliferation of VSMCs under high glucose culture [37]. Moreover, MAPK signaling pathway may be involved in the effect of high glucose on VSMC apoptosis [19]. ERK1/2 plays an important role in cell proliferation, differentiation and cell survival. It has been reported that hyperglycemia (25 mM glucose conditions) can promote the activation of ERK 1/2, JNK/SAPK or p38 signaling proteins in VSMCs of porcine and rat thoracic aorta [38, 39]. Further, activation of ERK1/2 MAPK signaling pathway can promote the proliferation and migration of VSMCs, as well as inhibiting their apoptosis under high glucose condition [39–41].

Glucagon-like peptide-1 (GLP-1) is a gut incretin, and modulates glucose-dependent insulin secretion and suppresses the release of glucagon. GLP-1 analogues and DPP-4 inhibitors has been used for the treatment of diabetes mellitus by increasing GLP-1 concentrations in the blood [42]. Besides its hypoglycemic effects, studies have revealed a potential cardioprotective effect of GLP-1 [43], *In vitro* experiments have demonstrated that exendin-4, a GLP-1 receptor agonist, may significantly reduce platelet-derived growth factor (PDGF)-induced proliferation of VSMCs [44]. DPP-4 inhibitors have been found to increase active GLP-1 concentrations by 2-3-fold in type 2 diabetes [45]. Furthermore, the DPP-4 inhibitors des-fluoro-sitagliptin and alogliptin, have been reported to exert favorable effects in atherosclerosis [10, 46]. Matsubara *et al.* demonstrated that DPP-4 inhibitors improved endothelial dysfunction and reduced the formation of atherosclerotic lesions in male apo E-deficient mice [46]. This study demonstrated that sitagliptin treatment inhibited the migration, proliferation and calcification and promoted apoptosis of HG-cultured VSMCs. Compared with previous studies, we focused on the functional examination of VSMCs under high glucose conditions in this study, which has certain clinical significance. Thus, sitagliptin, as a highly selective DPP-4 inhibitor, may act on VSMCs, thus promoting cardioprotective effects in diabetic patients. Such findings are of clinical importance: significant abnormalities in VSMCs are commonly found in diabetic patients with atherosclerosis and vascular calcification [24–26]. Inhibition of VSMCs migration, proliferation and calcification, and facilitation of apoptosis with DPP-IV inhibitors could prove a novel method in modulating vascular disease in patients with type 2 diabetes. However, confirmation of these findings will require further investigation in a clinical setting.

This study preliminarily explored the mechanism of cardiovascular protective effects of sitagliptin. Previous studies have shown that sitagliptin can inhibit the proliferation and migration of VSMCs induced by PDGF, tumor necrosis factor alpha (TNF-α) or DPP-4, suggesting that sitagliptin may achieve its effects on VSMCs through its anti-inflammatory action and inhibition of DPP-4[47]. In this study, HG stimulation triggered the up-regulation of the ERK signal pathway in VSMCs, which could be reversed by pretreatment with sitagliptin, observed as reduced phosphorylation levels in VSMCs. Combined with the finding that VSMCs exhibited a decrease in proliferation, migration and calcification, and an increase in cell apoptosis in the presence of the ERK1/2 inhibitor PD98059, this study revealed the potential involvement of ERK1/2 signaling pathways in the prevention of HG-induced alterations of VSMC migration, proliferation, calcification and apoptosis by pretreatment with sitagliptin.

However, there are some caveats to our findings. In this study, as we did not determine the cellular concentration of GLP-1, it is difficult to judge whether the effects of sitagliptin on VSMCs were GLP-1-dependent, a question that may be addressed in future studies. Besides GLP-1, some unmeasured DPP-4 substrate chemokines or proteins, such as SDF-1α, peptide YY and brain natriuretic peptide (BNP) may also influence the observed effects of sitagliptin on VSMCs, which requires further investigation.

In conclusion, this study showed that HG treatment facilitated migration, proliferation and calcification of VSMCs and inhibited their apoptosis. These effects could be attenuated by pretreatment with sitagliptin, most likely through the inhibition of ERK1/2 signaling pathways. The beneficial effects of sitagliptin on VSMCs exposed to HG reveal that sitagliptin may be an effective therapeutic option in the prevention of diabetic atherosclerosis.

## MATERIALS AND METHODS

### Animals

Male Sprague Dawley rats (n = 4; 5–8 wks) were provided by the Laboratory Animal Center of Harbin Medical University, China. All procedures were performed in accordance with the guidelines set by the Institutional Animal Care and Use Committee of the First Affiliated Hospital of Harbin Medical University, which is in compliance with the Animal Research: Reporting of *In Vivo* Experiments (ARRIVE) guidelines on animal research [48].

### Reagents

Sitagliptin was purchased from Santa Cruz Biotechnology (Santa Cruz, CA, USA). The ERK1/2 inhibitor (PD98059) and FITC-conjugated anti-α-smooth muscle actin (α-SMA) monoclonal antibody were obtained from Sigma-Aldrich (St Louis, MO, USA). Mouse monoclonal core-binding factor α 1 (cbfa1) (ab54868) and mouse monoclonal α-SMA antibody (ab7817) were obtained from Abcam (Cambridge, MA, USA). Trypsin, fetal bovine serum (FBS) and Dulbecco's Modified Eagle's Medium (DMEM) were purchased from Gibco (of Thermo Fisher Scientific, Waltham, MA, USA). Rabbit polyclonal antibody against ERK1/2 and a mouse monoclonal antibody against p-ERK1/2 were purchased from Santa Cruz Biotechnology Inc. (Dallas, TX, USA). Secondary antibodies were purchased from Vector Labs (Burlingame, CA, USA). Western blot bands were detected using the ECL Advance Western blotting detection kit from GE Healthcare (Chalfont St Giles, UK). Cell counting kit-8 was purchased from Dojindo Molecular Technologies (Rockville, MD, USA). Transwell plates were purchased from Millipore (Bedford, MA, USA). Annexin V-FITC Kit was purchased from BD Biosciences (San Jose, CA, USA). The alkaline phosphatase (ALP) activity kit was from Nanjing Jiancheng Bioengineering Institute (Nanjing, China). Calcium Colorimetric Assay Kit was obtained from Biovision (Mountain View, CA, USA).

### Cell culture

VSMCs were prepared from the thoracic aorta of Sprague Dawley rats as [27, 49, 50] previously described, with minor modifications [41] [51]. Whole thoracic aorta was isolated from sacrificed rats, and the endothelial cells were removed with a sterile toothpick inserted into the vascular lumen back and forth twice. The vascular adventitia was carefully stripped with ophthalmic tweezers. The thoracic aorta was then cut open longitudinally and rinsed with PBS solution twice. The aortic tissue was cut into small pieces (1 mm^2^), which were then plated into a tissue culture flask and cultured in DMEM supplemented with 15 % FBS incubated at 37 °C and 5 % CO2. The cells were passaged by trypsinization and reseeded into new flasks approximately 4–8 times before use in subsequent experiments. VSMCs were identified by their specific biomarker α-SMA. Cells at 4 to 8 passages were used for *in vitro* experiments.

### Cell treatment protocol

After an initial 24 h of culture in serum-free medium, the VSMCs were subjected to: 1) Control medium containing 5 mM glucose (control group); 2) Osmotic medium containing 20 mM mannitol (MG); 3) High glucose (HG) medium containing 25 mM glucose [27, 50, 52] (HG group); 4) Treatment with 200 nM sitagliptin in control medium (SITA group); 5) treatment with 200 nM sitagliptin in HG medium (HG+SITA group); 6) the ERK1/2 inhibitor PD98059 (50 μM) in HG medium (HG+PD98059 group). VSMCs were pretreated with the indicated agents for 1 h before culture in HG condition. For long-term experiments, the sitagliptin was added every two days when the culture media were changed.

### Cell proliferation assay

Cells were plated in 96-well culture plates and cultured until they reached 80 % confluency. Then, the cells were cultured with serum-free DMEM for 24 h and then were divided according to designated groups for culture for 12, 24, 48 h. Cell proliferation was then assessed using a Cell Counting Kit-8 according to the manufacturer's instructions. Briefly, the colorimetric reagent, 2-(2-methoxy-4-nitrophenyl)-3-(4-nitrophenyl)-5-(2,4-disulfophenyl)-2H-tetrazolium monosodium salt, was added to each sample and incubated for 1 h at 37 °C. The reaction was then assessed by measuring absorbance of each sample at a wavelength of 450 nm.

### Transwell migration assay

Cell migration was determined using the Transwell assay as previous described, with minor modifications [53]. Briefly, VSMCs were treated according to their designated group for 12 h, 24 h at 37 °C. Cells were trypsinized with 0.25 % (v/v) trypsin and re-suspended in serum-free DMEM at 37 °C. These cells were then counted and seeded in the upper chamber of each Transwell at a concentration of 1 × 10^5^ cells in 0.2 mL serum-free DMEM. 0.8 mL of DMEM supplemented with 20 % FBS [20, 54–56] was added to the lower chamber of each Transwell. Chambers were incubated for 12 h at 37 °C with 5 % CO_2_. Cells that migrated to the underside of the Transwell filter were fixed with 4 % formaldehyde (w/v) for 20 min at room temperature and then stained with hematoxylin. The staining was examined by microscopy at 200× magnification.

### *In vitro* scratch wound assay

The migration capacity of VSMCs was determined using an *in vitro* scratch wound model [56, 57]. Briefly, after VSMCs grown to confluence, the cells were cultured with serum-free DMEM for 24 h, and then a scratch wound was made using a 200 μL sterile pipette tip. The VSMCs were then continually cultured for another 24 h in the presence of the indicated treatment(s) as mentioned above. The scratch wounds were observed at 0 and 24 h, and the capacity of VSMCs to migrate was evaluated by measuring the width of the scratch wound at both time points using ImageJ [58].

### Assessment of cell apoptosis

Cell apoptosis was measured using the Annexin V-FITC kit according to the manufacturer's instructions. Briefly, the cells were cultured with serum-free DMEM for 24 h, and then cells were treated according to their designated group for 48 h and then harvested by trypsinization. After washing, cells were then collected and re-suspended in 500 μL of binding buffer, and stained with 5 μL of Annexin V-FITC and 5 μL of propidium iodide solution for 15 min at room temperature in the dark. The percentage of Annexin V-FITC- and propidium iodide-positive cells was measured by flow cytometry (FACSAria, BD Biosciences, San Jose, USA).

### Western blot analysis

Cells were collected and lysed in 200 μL of radioimmunoprecipitation assay buffer with the protease inhibitor phenylmethylsulfonyl fluoride (100 mM) for 1 h on ice. After centrifugation, the supernatant was collected and the total protein concentration was determined using a bicinchoninic acid kit (Thermo Fisher Scientific, Cleveland, USA). 10 μg of total protein from each sample was separated by electrophoresis using 12 % SDS-PAGE gels and transferred onto nitrocellulose membranes. The membranes were incubated with primary antibodies (1:10,000) and then their corresponding secondary antibodies (1:50,000). The density of each band was measured using Quantity One 4.62 (Bio-Rad Laboratories, Inc., Hercules, CA, USA) and corrected by reference to the value of β-actin. Levels of phosphorylated proteins were determined as a ratio of total protein: p-ERK1/2 relative to ERK1/2.

### Alizarin red staining

Cells were fixed in 70 % ethanol for 1 h at room temperature and stained with 40 mM Alizarin Red S for 10 min. Cells were washed twice with PBS and the staining was eluted by adding cetylpyridinium chloride per well for 30 min at room temperature. The supernatants were collected and detected by a spectrophotometer at 562 nm.

### Measurement of alkaline phosphatase activity (ALP) activity

On the third, seventh and fourteenth day of culture, the cultured cells was added to 100 μL of PBS, followed by repeated freezing and thawing in liquid nitrogen for three times. After centrifugation at 12000 rpm for 10 min, the supernatant was carefully collected and stored at -20 °C. Protein quantification was performed using the standard curve prepared. ALP activity was measured using an ALP activity kit according to the manufacturer's instructions.

### Statistical analysis

SPSS for Windows version 17.0 software package (SPSS Inc., Chicago, IL) was used for the statistical data analysis. Results were expressed as mean ± standard deviation (SD) from three independent experiments. Data were analyzed using one-way analysis of variance (ANOVA). *P*<0.05 was considered statistically significant.

## Abbreviations

ALP: Alkaline phosphatase
AMI: Acute Myocardial Infarction
Bcl-2: B-cell Lymphoma 2
Bcl-xL: B-cell Lymphoma xL
Bfl-1/A1: Bcl-2-related Protein A1
BMP-2: Bone morphogenetic protein-2
Cbfα-1: Core Binding Factor α1
DM: Diabetes mellitus
DMEM: Dulbecco's Modified Eagle's Medium
DPP-4: Dipeptidyl Peptidase-4
ERK: Extracellularsignal-regulated kinase
FBS: Fetal Bovine Serum
GLP-1: Glucagon-like peptide
HG: High glucose
MAPK: Mitogen-Activated Protein Kinases
PDGF: Platelet-derived growth factor
ROS: Reactive Oxygen Species
SM-α-actin: Smooth Muscle alpha-actin
TNF-α: Tumor necrosis factor alpha
VSMC: Vascular smooth muscle cell
